# Tobacco retail characteristics across urban and rural districts in Lao PDR: an observational study, 2024

**DOI:** 10.3389/fpubh.2026.1784257

**Published:** 2026-03-24

**Authors:** Shweta Kulkarni, Thanh Cong Bui, Phonepadith Xangsayarath, Khatthanaphone Phangdouangsy, Chanthavy Soulaphy, Khanittha Sengdara, Sydney A. Martinez, Amanda Janitz, Summer G. Frank-Pearce, Laura A. Beebe

**Affiliations:** 1 Department of Biostatistics and Epidemiology, Hudson College of Public Health, University of Oklahoma, Oklahoma City, OK, United States; 2 Department of Family and Preventive Medicine, College of Medicine, University of Oklahoma, Oklahoma City, OK, United States; 3 TSET Health Promotion Research Center, Stephenson Cancer Center, University of Oklahoma, Oklahoma City, OK, United States; 4 Department of Communicable Disease Control, Ministry of Health of Lao People's Democratic Republic (Lao PDR), Vientiane, Laos; 5 Secretariat of the National Tobacco Control Committee, Department of Hygiene and Health Promotion, Ministry of Health of Lao PDR, Vientiane, Laos

**Keywords:** Lao PDR (Laos), public health, tobacco advertising and promotion, tobacco control, tobacco retail environment

## Abstract

**Introduction:**

Exposure to tobacco advertising and promotion is linked to smoking initiation among youth. Very little is known about the tobacco retail environment in the Lao People’s Democratic Republic (PDR). This study examines the characteristics of tobacco retailer outlets (TROs) and tobacco advertising and promotion in three districts of the Lao PDR capital city Vientiane, two urban (Sissatanak and Chanthabuly) and one rural districts (Naxaithong).

**Methods:**

In each district, we defined a catchment area to identify TROs around schools (500 meters in urban and 1,000 meters in rural districts). We conducted the audit between January 19 and February 18, 2024. Observers assessed and recorded TRO types, cigarette sales, and tobacco advertisements and promotions.

**Results:**

A total of 233 TROs were audited around schools: 113 in Sissatanak, 90 in Chanthabuly, and 30 in Naxaithong. TRO types included convenience stores (40.3%), supermarkets (27.9%), roadside kiosks (17.2%), and restaurants (6.0%). TROs in the urban districts, Sissatanak (38.6%) and Chanthabuly (48.5%) were more likely to display age verification signs compared to the Naxaithong (12.8%) (*p* value<0.05). Also, Naxaithong had more outside advertisements (90.0%) compared to both urban districts (54.7%) (*p* value <0.001). Tobacco advertisements were more common in Naxaithong (90.0%) than in Chanthabuly (51.3%, *p* value<0.01) or Sissatanak (58.0%, *p* value<0.01). Supermarkets had the highest proportion of age verification signs (29.2%) compared to all the other TRO types (*p* value<0.01).

**Conclusion:**

Our study provides cross-sectional evidence on tobacco retail outlet characteristics around schools in urban and rural districts of Vientiane, Lao PDR. We observed variation in retail characteristics, including a higher prevalence of outdoor tobacco advertisements and in-store branding materials in rural districts compared with urban districts.

**Implications:**

Our study provides cross-sectional evidence on the tobacco retail environment in Vientiane, Lao PDR. Based on the observations and primary data collected in January–February 2024, our study found continued retail-level promotional practices, limited age-verification signage, availability of low-priced cigarettes, and variability in compliance with tobacco control regulations across retail outlet types. While these findings are descriptive in nature, they suggest that strengthened monitoring and enforcement of existing regulations may support more effective implementation of tobacco control policies in Lao PDR.

## Introduction

The tobacco retail environment plays an important role in shaping smoking behaviors including smoking initiation, cessation, and relapses. Studies have consistently shown that the availability, accessibility, and marketing of tobacco products in tobacco retail outlets (TROs) can influence an individual’s smoking behavior (1). TROs have a central role to play in the tobacco industry marketing as product display, pricing, placement and promotion all occur in one place (2). Point-of-sale advertising near schools has been documented in Indonesia (3), and school-aged children in the Philippines and Indonesia have reported receiving tobacco product promotions despite bans (4). Thus, cross-sectional observation of tobacco retail environment reveals tobacco industry marketing tactics specifically designed to target youth and understand the extent of implementation of local tobacco control policies.

The Association for Southeast Asian Nations (ASEAN) consists of ten countries: Brunei Darussalam, Cambodia, Indonesia, Lao People’s Democratic Republic (PDR), Malaysia, Myanmar, the Philippines, Singapore, Thailand, and Vietnam (5). Tobacco use causes more than half a million deaths annually in the region, with Lao PDR having one of the highest adult smoking rates - 51% among men, second only to Indonesia (63%) and 7.1% among women, the highest in the ASEAN (6). The tobacco industry in the ASEAN often circumvents tobacco advertising restrictions through “surrogate advertisement,” a tactic that exposes consumers to tobacco branding without showing direct advertisements (7). Despite the high smoking prevalence and presence of marketing tactics by tobacco industry, research on tobacco retail environment in the region is limited. This study addresses this research gap by presenting observational evidence on tobacco retail characteristics in Lao PDR, a country with one of the highest smoking prevalence in the ASEAN.

Observational studies across ASEAN have documented tobacco advertisements near schools in Indonesia (8), violations of advertising bans among youth in Vietnam (9), and associations between tobacco advertising, promotion, and sponsorship (TAPS) and high school smoking in Myanmar (10). Evidence also shows that tobacco retail outlets (TROs) are highly concentrated around schools, with high outlet density in Thailand (11), minimal enforcement of youth sales restrictions in Indonesia (2), and greater retailer density near children’s facilities in rural versus urban areas of Indonesia (12). The observational evidence on TRO characteristics near schools in Lao PDR is scarce. This study addresses this gap by systematically examining the tobacco retail environment near schools in urban and rural areas of Lao PDR, providing critical cross-sectional evidence on the implementation of local tobacco control policies.

Lao PDR ratified the World Health Organization Framework Convention on Tobacco Control (WHO FCTC) in September 2006, the first international, evidence-base treaty developed to address the globalization of tobacco epidemic (13). But the enforcement of tobacco control policies is not consistent across the country. Lao PDR’s Ministry of Health mandated all tobacco companies to print health warnings covering 75% of cigarette packaging, in 2016 (14), however, a market analysis in 2018 found that most popular cigarette brands frequently violated this requirement (15). Lao PDR made some progress in restricting TAPS in 2021, prohibiting tobacco product advertising at the point of sale (POS), and promotional discounts (16), but previous research on tobacco retailers found widespread availability of cigarettes with low prices (< 1 US$) at POS (17). We conducted a systematic and cross-sectional observation of tobacco retail environment to assess the extent of implementation of local tobacco control policies. The present study aims to examine the characteristics of TROs including the presence of tobacco advertising and promotions at TROs in three districts of Vientiane, the capital city of Lao PDR.

## Methods

### Study design

We physically canvased three districts within Vientiane and conducted observational surveys to collect data on tobacco retail characteristics in the catchment area described below.

We conducted an observational study in two urban districts (Chanthabuly and Sissatanak) and one rural district (Naxaithong) within Vientiane between January 19 and February 18, 2024. Chanthabuly and Sissatanak have population densities of 2,971/km^2^ and 2,831/km^2^ respectively, while Naxaithong has a population density of 97.96/km^2^ as reported by the Lao Statistics Bureau (18). The study catchment area was defined using the following strategy.

### Sampling strategy

#### Sampling frame

A list of licensed TROs in Lao PDR was not available for use as a sampling frame. Based on the results of a recent Global Youth Tobacco Survey in Lao PDR, adolescents of secondary school ages or above were the most vulnerable to smoking initiation (19). The age group 12 to 17 years represents the predominant age of smoking initiation among adolescents worldwide (12, 20). Thus, we defined a study catchment area around schools (secondary schools and above) in the three districts of Vientiane (Supplementary Figures 1, 2).

#### Sampling method

A two-stage cluster sampling approach with a non-random selection of clusters was used to identify the study area. In the first stage, two urban and one rural district were identified in Vientiane. The choice of the districts was governed by the population density of the districts (21), and based on logistical constraints related to time and resource availability.

In the second stage, a list of secondary schools and above within those three districts was obtained from the Lao Ministry of Education website and served as the sampling frame. Lower secondary schools serve ages 11–15 years and upper secondary schools ages 15–18 years. Schools with a completely documented address were selected. These criteria identified seven schools in Chanthabuly, five in Sissatanak, and six schools in Naxaithong.

We additionally included a convenience sample of nine schools selected based on: (i) proximity to the main study site, the National Center for Laboratory and Epidemiology in Vientiane, and (ii) availability of a complete, documented address provided by local research coordinators. The inclusion of these nine schools was necessary because not all schools listed on the Ministry of Education website could be reliably mapped using publicly available street address information. Furthermore, time and resource constraints required us to limit the number of schools listed with Ministry of Education, that could be included in the study. Additionally, some private/ international schools located in urban districts were not registered with the Ministry of Education; therefore, a convenience sample of nine additional schools was included to ensure their representation.

These processes were identified in a total of 27 selected schools (11 schools in Chanthabuly, 10 schools in Sissatanak, and six schools in Naxaithong) that formed the catchment area for TROs (Supplementary Table 1). Data on school characteristics were also obtained including levels of school (secondary school, high school, college), total number of students per school, and the annual fee structure of the school. Since the schools in the urban and rural districts differed from each other based on fee structure and number of students per school, we described the characteristics of each school in our study (mean number of students per school and mean annual fee structure).

#### Justification of the catchment area

A 500-meter radius was defined and canvased around each school in the two urban districts, while in the rural district, a radius of 1 km defined the catchment area. A larger radius was chosen for the rural district since schools in rural districts are located at considerable distances from each other (22). These distances, i.e., 500 m and 1 km, represent feasible catchment areas and have been used in prior studies (2, 12).

#### Selection criteria for TROs

All TROs visible from the streets or alleys were audited and mapped. TROs were defined as any store that sold consumer goods, and included kiosks, restaurants and food vendors, street vendors, mini markets, and supermarkets. We included TROs on the first floor of malls, traditional markets, and shopping centers if they were visible from the street but excluded any that were not readily visible from the street.

### Data collection tools

#### Design of the Tobacco Audit Survey

We compiled the Tobacco Audit Survey based on the Standardized Tobacco Assessment for Retail Settings (STARS), a validated surveillance tool used by practitioners in the US to inform state and local tobacco control for the POS (specifically questions relating to ‘product, promotion, and price’) (23). Another validated questionnaire, the Environmental Profile of a Community’s Health (EPOCH) survey (24), was also used, specifically for questions relating to the presence of PHWs, and the number of cigarettes sold per pack. In other studies, the EPOCH survey collected data on tobacco policy implementation among those low- and middle-income countries that have ratified the WHO FCTC (24). We added questions related to ‘targeting youth’ and ‘types of cigarette brands sold’ specific to the Lao PDR context and the study methodology of auditing shops around schools. Study data were collected and managed using REDCap electronic data capture tools hosted at the University of Oklahoma Health Sciences (25, 26). We ensured that the pictures taken during the audit were of high quality and clarity, each picture taken had a unique label corresponding to the audited TRO. After entering data on REDCap, we used the Data Comparison Tool to identify any discrepancies which were resolved during data export.

The survey included four main sections namely Product, Promotion, Price, and Targeting Youth. The Product section assessed five items: (1) presence of PHWs on cigarette packs (Yes/No), (2) number of brands of cigarettes sold, (3) number of cigarettes in a single cigarette pack, (4) presence of a store staff/owner inside the store, and (5) seeing anyone smoke inside or outside the store. The Promotion section asked for two items: (1) the presence of cigarette advertising (Yes/No), and (2) the presence of branded material placed inside or outside the store. The Price section asked for three items: (1) the name and price of cigarette brands being sold, (2) the availability for sale of a single loose cigarette, and (3) the name and price of the cheapest brand of cigarette. Finally, the section on Targeting Youth assessed two items: (1) the presence of tobacco products within 12 inches of candies, gums, cold drinks, and ice cream (Yes/No), and (2) the display of tobacco products within three feet of the floor (Yes/No).

#### Other measures

Data on TRO types were categorized into convenience stores, supermarket or grocery stores, roadside kiosks, restaurants having informal outlets, and others which comprised all other businesses selling tobacco products. The data on the cheapest brand of cigarettes were categorized as ‘Bastos Red, Bastos Green, and Others’ and data on the price of the cheapest cigarette brand (in Lao Kip- LAK) were categorized as 4,000-7,000, 7,001–8,000, 8,001-10,000, and >10,000.

#### Global positioning system (GPS) data collection

GPS data regarding the coordinates of the schools and tobacco shops were obtained using the Garmin GPSMAP 67i portable GPS device.

### Data collection procedures

#### Audit procedure of TROs

The audit area was covered by visiting each of the selected schools and traveling along roads within the respective 500 m and 1 km catchment zones for urban and rural districts. Audits consisted of passive observation by the data collectors and were conducted over 1 month from January 19 to February 18, 2024, by the primary author (SK) and two local data collectors. The team walked and used private transportation to cover the streets and alleys in the sampled area around the schools. The geocoordinates of schools and TROs were recorded with a precision of ≤ 10 meters. We observed all forms of tobacco advertising including banners, posters, billboards, video advertisements, outdoor advertisements on stores, stickers, merchandise, and any other items containing tobacco brand logos that were visible from the street and in all areas of the retail shops (27).

Observations occurred between 9 a.m. – 6 p.m. to match the opening hours of most TROs. Digital photographs of the tobacco products inside TROs were taken. The team conducted a mock audit of the TROs a few days before data collection to ensure a complete understanding of the audit protocol. The training consisted of field demonstrations of identifying TROs and advertising types, exercise in walking routes to ensure coverage of catchment zones, and inter-observer reliability checks. The inter-observer reliability checks consisted of team members auditing around 10% of the sampled TROs (~ 23–25 TROs) and comparing the resulting to ensure consistency in recording the data and classifying into correct TRO type.

### Analysis

#### Statistical analysis

Given the observational and cross-sectional design of this study, analyses were conducted to describe and compare differences in the observed TRO characteristics at a single time point. We performed a Chi-square test to examine if there is a significant differences in the distribution of TRO categories across the three districts, compared together. We also performed a Fisher’s Exact test to examine differences in the presence of age verification at TROs, sale of alcoholic beverages, and cigarette pack characteristics (presence of PHWs, presence of cigarette advertisements, and branding material) comparing each urban district to the rural district. Similarly, we performed analysis for other TRO characteristics including the sale and price of cigarette packs, and the proximity of tobacco products to other products with potential appeal to youth. We performed the Fisher’s Exact test to examine the differences in the cheapest brand of cigarette sold, its price, and the sale of tobacco products near other youth-friendly products across the urban and rural districts. Similarly, we examined whether each of the TRO characteristics differed across the categories of TRO outlets observed in our study. SAS 9.4 was used to perform the above analyses and a *p* value of ≤ 0.05 was considered statistically significant. We used bar graphs to present the different types of TRO outlets across the three districts.

#### Geospatial analysis

The GPS data were cleaned and converted to DMS (degrees, minutes, seconds) format and later imported onto the ArcGIS Pro software. We presented the distribution of the 27 schools across the three districts (Supplementary Figure 1).

Our study was determined to not meet the criteria for human subjects by the Institutional Review Board, Human Subject Determination, University of Oklahoma Health Sciences (IRB # 16649).

## Results

We audited 233 TROs around 11 schools in Sissatanak, 10 schools in Chanthabuly, and 6 schools in Naxaithong. Naxaithong had the highest mean number of students per school, while Sissatanak had the highest mean annual fee structure (4,101,300 LAK) followed by Chanthabuly (1,087,710 LAK) and Naxaithong (306,084 LAK). The distribution of the schools across the three districts is shown in Supplementary Figure 1.

**Figure 1:**
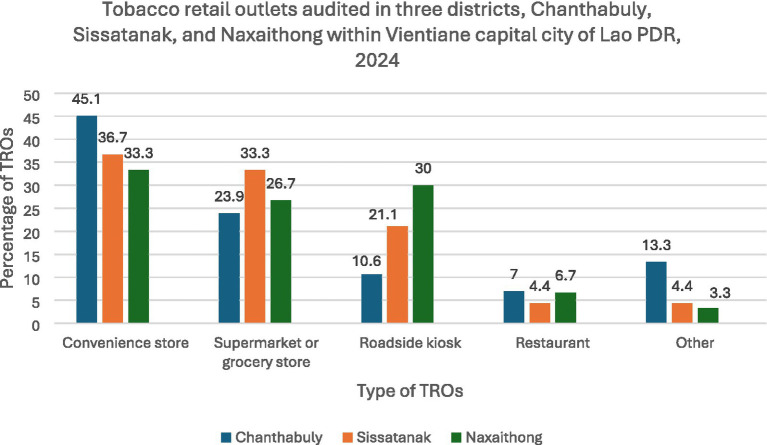
Types of tobacco retail outlets audited across three districts namely Chanthabuly, Sissatanak, and Naxaithong, within Vientiane the capital city of Lao PDR, 2024. *p* value for the Chi-squared test = 0.046.

The descriptions of each of the TRO categories observed in our study are provided in Supplementary Table 2. The convenience store was the most common type of TRO (40.0%) followed by the supermarket or grocery store (27.9%), roadside kiosk (17.2%), and restaurant (6.0%). There was a significant difference in the TRO types between the three districts. Chanthabuly district had the highest proportion of convenience store TROs (45.1%), while Naxaithong had the highest proportion of roadside kiosks (30.0%) (Figure 1).

Age verification signage was found in only the two urban districts of Chanthabuly (15.9%) and Sissatanak (7.8%) but not in Naxaithong. A significantly lower proportion of TROs in Chanthabuly (69.9%) had PHWs on cigarette packs compared to Naxaithong (90.0%) (*p* value = 0.033) but the difference was not significant when comparing Sissatanak (84.4%) with Naxaithong (90.0%). Additionally, cigarette advertisements (placed outside) and branding materials (placed inside) were more frequent in the rural district of Naxaithong compared to either Chanthabuly (*p* value < 0.05 for both advertisements and branding materials) or Sissatanak (both *p* values < 0.05). We observed a high proportion of sales of alcohol along with tobacco products in all three districts (Table 1).

**Table 1:** Tobacco retail outlet characteristics and tobacco products and promotion: comparing each of two urban districts with a rural district of Vientiane, capital city of Lao PDR, 2024.

Tobacco retail outlet characteristics	Chanthabuly (urban) vs. Naxaithong (rural districts)			Sissatanak (urban) vs. Naxaithong (rural districts)		
	Chanthabuly (*N*, %)	Naxaithong (N, %)	Chi-squared test*, p* value	Sissatanak (*N*, %)	Naxaithong (*N*, %)	Chi-squared test*, p* value
Number of tobacco shops audited	113 (48.5)	30 (12.8)		90 (38.6)	30 (12.8)	ND
Age Verification displayed inside shop (purchase of tobacco products for those aged 18 years and above)	18 (15.9)	0	0.013*	7 (7.8)	0	0.190*
Sale of alcoholic beverages (including beer, wine, or liquor) alongside tobacco products	106 (93.8)	28 (93.3)	0.258	85 (94.4)	28 (93.3)	0.775
Presence of health warning images on the cigarette packs	79 (69.9)	27 (90.0)	**0.033**	76 (84.4)	27 (90.0)	0.557
Presence of a store staff/owner inside the store	112 (99.1)	30 (100.0)	0.790	88 (97.8)	30 (100.0)	0.560
Cigarettes advertised ‘outside’ the store	58 (51.3)	27 (90.0)	**<0.001**	53 (58.9)	27 (90.0)	**<0.001**
Branding material for cigarettes placed ‘inside’ the store	12 (10.6)	8 (26.7)	**<0.036**	7 (7.8)	8 (26.7)	**<0.011**
Number of cigarette brands sold in the store (Mean, standard deviation); (Range)	4.2 (1.8) (2, 10)	4.6 (2.2) (2, 10)	<0.001**	3.8 (1.4) (2, 9)	4.6 (2.2) (2, 10)	0.338**

N (Total number of Tobacco Retail Outlets audited) = 233; *p* value ≤ 0.05 was considered significant and marked in bold; *p value for Fisher’s Exact test since the cell count is less than 5, ** *p* value for t-test; Reference group for tobacco retail characteristics is the ‘absent’ category.

The cigarette brands ‘Bastos Red’ and ‘Bastos Green’ were the cheapest brands sold in Chanthabuly (*p* value = 0.013) and Sissatanak (*p* value = 0.002) when compared to the rural districts. When compared individually to Naxaithong, Chanthabuly had the highest proportion of cigarette packs (32.7%) sold for the price range 8,001–10,000 LAK (*p* value <0.001 compared to other pack prices), while Sissatanak had the highest proportion of cigarette packs (35.6%) sold for the price range 7,001–8,000 LAK (*p* value <0.001 compared to other pack prices) (Table 2).

**Table 2:** Sale and price of cigarettes, and targeting youth: comparing each of two urban districts with a rural districts of Vientiane, capital city of Lao PDR, 2024.

Tobacco retail outlet characteristics	Chanthabuly (urban) vs. Naxaithong (rural districts)			Sissatanak (urban) vs. Naxaithong (rural districts)		
	Chanthabuly (*N*, %)	Naxaithong (*N*, %)	*p* value**	Sissatanak (*N*, %)	Naxaithong (*N*, %)	*p* value**
Number of tobacco shops audited	113 (48.5)	30 (12.8)		90 (38.6)	30	ND
Sale of ‘loose’ cigarette	2 (1.8)	1 (3.3)	0.648	1 (1.1)	1 (3.3)	0.489
Name of the cheapest brand of cigarette						
Bastos Red	56 (51.4)	6 (20.0)	**0.013**	29 (34.1)	6 (20.0)	**0.002**
Bastos Green	43 (39.5)	15 (50.0)		51 (60.0)	15 (50.0)	
Other*	10 (9.2)	9 (30.0)		5 (5.9)	9 (30.0)	
*Missing (N = 7)*						
Price of the cheapest cigarette brands sold						
4,000–7,000 LAK (< 0.33 USD)	30 (26.5)	0	**<0.001**	23 (25.6)	0	**<0.001**
7,001–8,000 LAK (0.33–0.38 USD)	34 (30.0)	5 (16.7)		32 (35.6)	5 (16.7)	
8,001–10,000 LAK (0.38–0.47 USD)	37 (32.7)	17 (56.7)		25 (27.8)	17 (56.7)	
>10,000 LAK (>0.47 USD)	12 (10.6)	8 (26.7)		10 (11.1)	8 (26.7)	
Tobacco products sold within 12 inches of candy, gum, cold drinks, or ice cream	108 (78.3)	30 (21.7)	0.302	88 (74.6)	30 (25.4)	0.560
*Missing (N = 7)*						
Tobacco product advertisements displayed within 3 feet of the floor	110 (78.6)	30 (21.4)	0.490	88 (74.6)	30 (25.4)	0.560
*Missing (N = 5)*						

N (Total number of Tobacco Retail Outlets audited) = 233; *p* value ≤ 0.05 was considered significant and marked in bold, *Other included cigarette brands such as JN Green, Lao Tobacco Red, Bastos Black, Bastos Blue, JN Blue, and Chinese brand of cigarettes; ** *p* value for Fisher’s Exact test.

There were significant differences in age verification signage inside TROs, sale of alcoholic beverages alongside tobacco products, presence of PHWs on cigarette packs, sale of ‘loose’ cigarettes, and cigarette advertisements based on the type of TRO outlet when all districts were combined (Table 3). Supermarket or grocery store-type TROs had the highest proportion of age verification signage displayed inside (29.2% compared to all other TROs; *p* value <0.01). The presence of PHWs on cigarette packs was observed more frequently in TROs attached to a restaurant (85.7%) (*p* value = 0.014). Roadside kiosks were more likely to sell single cigarettes (7.5%) (*p* valu*e* <0.01) and had the highest proportion of display of cigarette advertisements (82.6%) (in the form of displaying the cigarette packs in transparent cabinets) (*p* value <0.01) (Table 3).

**Table 3:** Tobacco retail outlet characteristics by type of retail outlet in Lao PDR, 2024.

Tobacco retail outlet (TROs) characteristics	Types of tobacco retail outlets (*N* = 233)					
	Convenience store	Supermarket or grocery store	Roadside kiosk	Restaurant	Other	*p* value*
	*N* = 94	*N* = 65	*N* = 40	*N* = 14	*N* = 20	
Age verification signage displayed inside shop (purchase of tobacco products for those aged 18 years and above)	2 (2.1)	19 (29.2)	1 (2.5)	1 (7.1)	2 (10.0)	**<0.001**
Sale of alcoholic beverages (including beer, wine, or liquor) alongside tobacco products	94 (100.0)	61 (93.8)	35 (87.5)	14 (100.0)	15 (75.0)	**<0.001**
Presence of health warning images on the cigarette packs	75 (79.8)	54 (83.0)	32 (80.0)	12 (85.7)	9 (45.0)	**0.014**
Cigarettes advertised ‘outside’ the store	56 (59.6)	28 (43.0)	33 (82.5)	11 (78.6)	10 (50.0)	**<0.001**
Sale of ‘loose’ cigarette	0	0	3 (7.50)	0	0	**<0.001**
Tobacco products sold within 12 inches of candy, gum, cold drinks, or ice cream	93 (98.9)	64 (98.5)	39 (97.5)	13 (92.9)	NA	**<0.001**
Tobacco product advertisements displayed within 3 feet of the floor	93 (98.9)	64 (98.5)	39 (97.5)	13 (92.9)	17 (85.0)	**<0.001**

N (Total number of tobacco retail outlets audited) = 233; *p* value ≤ 0.05 was considered significant and marked in bold; *p value for Fisher’s Exact test examining if the proportion of tobacco retail shops with a specific characteristic differ significantly across the types of tobacco retail shops, tested separately; Reference group for tobacco retail characteristics is the ‘absent’ category.

The TRO pictures taken during the audits are presented in (Supplementary Figures 2A–C). We found that the tobacco products in TROs in our study were sold alongside common household items such as coffee, milk powder, eggs, and fruits. We also found that cigarette packs were organized neatly on cabinets near the store entrance and were sold in transparent cabinets. Most shops also sold lighters next to cigarette packs.

## Discussion

Our study found significant variations in the distribution of TRO types between urban and rural districts of Vientiane. Age verification requirements were present more frequently inside TROs in the urban districts, while the rural district had a higher prevalence of cigarette advertisements.

### Tobacco advertisements and point-of-sale product displays

*Under the Tobacco Control Law (as amended in 2021), all forms of tobacco advertising are prohibited in Lao PDR; however, ambiguity remains regarding the scope of permissible promotion at the point of sale (POS)* (28). Consistent with this gap, our study found cigarette packs often neatly arranged near store entrances, increasing their visibility and potential appeal to young customers. These practices resemble the “power wall” strategy, which includes prominent displays of cigarette brands, price cards, and branded materials typically located behind the cashier—which has been shown to function as a form of retail promotion (29). We found that supermarkets commonly sold tobacco products alongside youth-oriented items such as cold drinks, candies, gum, and ice cream, as well as everyday household goods including fruits, eggs, and coffee, representing a form of tobacco advertising and promotion through strategic placement and normalization. Although this study is cross-sectional and did not assess youth tobacco use directly, existing evidence has found association between POS displays, power walls and increased susceptibility of smoking among youth (28). Our findings therefore provide empirical evidence of ongoing retail-level promotional practices suggesting that stronger enforcements may be beneficial to limit tobacco product promotion at retail outlets in Lao PDR.

Our study identified outdoor tobacco advertisements at informal roadside kiosks, indicating potential violations of the Tobacco Control Law. These kiosks were also more likely to sell single cigarettes, a practice explicitly prohibited under Lao PDR law, *which bans the sale of single cigarettes and cigarette packs containing fewer than 20 sticks* (30). Similar findings have been reported in Bali, Indonesia, where kiosks were more likely to sell single cigarettes to youth (2). The sale of single cigarettes, as observed in our study, suggests non-compliance with the existing tobacco control regulation, highlighting gaps in enforcement of law.

### Age verification for tobacco purchases

*The Tobacco Control Law prohibits the sale of tobacco products to anyone under the age 18* (30). However, our study found a low proportion of age verification signage (purchase of tobacco products for those aged 18 years and above) in both urban and rural districts. Our finding is supported by evidence from Indonesia (2), and Myanmar (10) where tobacco sale were minimally discouraged among youth. Our study finding provides empirical evidence suggestive of inadequate age verification requirements at tobacco retail outlets in Lao PDR.

### Presence of pictorial health warnings on cigarette packs

*As of August 15, 2025, Lao PDR fully implemented standardized plain packaging with pictorial health warnings (PHWs) covering 75% of the front and back packaging* (31). We found a fewer proportion of cigarette packs having PHWs in urban districts compared to the rural district, on account of differences in cigarette brands sold across the districts. As our observations were conducted in 2024, prior to the full implementation of standardized plain packaging, continued monitoring could be beneficial to assess the extent and consistency of policy implementation over time.

### Tobacco excise tax rate

Despite high tax rates stipulated by law, tobacco taxes in practice remain low. Although the law mandates a 72% ad valorem tax plus LAK 800 per pack, the industry largely pays only a 15% tax, with LAK 200 per pack applied only to imports, leading to tobacco taxes accounting for just 18.8% of the retail price, among the lowest in the ASEAN region (32). Consistent with above policy context, our study found the presence of inexpensive cigarettes, with a higher proportion sold in rural districts compared to urban areas, potentially reflecting differences in the cigarette brands available across districts. Our finding is similar to evidence from Vietnam (33) and Indonesia (34) which found a variety of cigarette brands in the market with availability of economy cigarette brands. The cheapest cigarette brand, identified in our study, costed approximately 4,000 LAK (~0.19 USD) which is considerably lower compared to other ASEAN countries, including Thailand with a price of 0.4 USD/20-stick pack for the cheapest cigarette brand sold in 2020 (12), Myanmar with a price of 0.6 USD/20-stick pack in 2020 (10), and Malaysia with a price of 1.7 USD/20-stick pack in 2015 (35).

### Strengths and limitations

Our study provides the first cross-sectional evidence on the tobacco retail characteristics, near schools in urban and rural districts of Vientiane. Study findings were obtained using adapted, validated tools and standardized procedures to collect data on tobacco retail characteristics.

Our study findings might be biased due to systematic differences in TRO characteristics across districts and the three districts included in our study. However, our systematic approach to defining the catchment area using geographic coordinates and canvassing within that area ensured consistent coverage within the study area. The purposive selection of districts, while contextually justified, limits the generalizability and external validity of study findings beyond Vientiane. Tobacco retail outlet characteristics or sales patterns may differ across all districts due to variations in brand availability, pricing, or variations in enforcement of tobacco control regulation, public awareness or socio-economic conditions across districts. There is a possibility that the selected urban districts may have stricter implementation of national and provincial tobacco control policies (36) potentially introducing selection bias. As a result, the study findings may not fully represent the tobacco retail characteristics in other districts of Vientiane or in other provinces of Lao PDR. Future studies could more representative sample of tobacco retail outlets across larger and diverse areas of Lao PDR.

Similarly, the inclusion of convenience sample of schools (Supplementary Table 1) could potentially introduce selection bias in our findings. The schools included as a part of the convenience sample in the urban districts were primarily international or private schools, located in central urban areas whereas other schools were distributed more broadly across the urban districts. This sampling could result in differential exposure to tobacco retail types, such as, greater exposure to supermarkets and convenience stores and less to informal retail outlets, potentially overestimating observations related to tobacco control compliance in these retail types. Future studies could measure the differential exposure to retail characteristics based on school types (public Vs. private) and incorporate data from students on their exposure to various retail types. The opinions and expectations of data collectors could have influenced the audit process introducing an ‘observer bias’ wherein the data collectors might selectively record the data that aligns with their expectations or have systematic data recording errors. We attempted to avoid that bias by training data collectors, performing inter-observer reliability checks and using a structured data collection form.

Our study’s cross-sectional design limits the inference regarding temporal variability in tobacco retail practices or time-varying tobacco control enforcements. Future studies could include longitudinal or repeated audits across the sample, to potentially measure the effectiveness of tobacco control regulations on retail characteristics. Multivariable analyses were not conducted because this study was designed as descriptive cross-sectional observational study aimed at documenting and comparing tobacco retail characteristics across districts. Our sample size was limited, and we did not collect any data on the tobacco-purchasing behaviors of youth which constrained our ability to adjust for potential confounders. Consequently, our study findings only provide descriptive evidence on tobacco retail environment rather than evidence of the influence of tobacco retail environment on youth purchasing or use behaviors. Future studies could consider measuring youth exposure to POS advertisements or promotions, youth attitudes and consumption behaviors, and the influence of online advertising and cigarette pack branding (4).

Although our study collected geospatial data on the locations of schools and tobacco retail outlets across the three districts and conducted geospatial analyses, the present paper focuses on the descriptive, observational characteristics of tobacco retail outlets. Findings from the geospatial analyses will be reported in a separate publication.

## Conclusion

Our study provides cross-sectional evidence on tobacco retail outlet characteristics around schools in urban and rural districts of Vientiane, Lao PDR. We observed variation in retail characteristics, including a higher prevalence of outdoor tobacco advertisements and in-store branding materials in rural districts compared with urban districts. Despite the presence of comprehensive tobacco control policies in Lao PDR, our observations suggest continued retail-level promotional practices, limited age-verification signage, the availability of low-priced cigarettes, and variability in compliance with tobacco control regulations across retail outlet types. While these findings are descriptive in nature, they suggest the need for strengthened monitoring and enforcement of existing regulations for more effective implementation of tobacco control policies in Lao PDR.

### Future directions

There is limited evidence on monitoring and inspection of tobacco control policies between urban and rural districts areas of Lao PDR. While some urban areas of Lao PDR have implemented interventions to enforce retailer-level tobacco control regulations, such as targeted pilot intervention for retailer engagement (37), there is no evidence of similar initiatives in rural districts areas. Additionally, global evidence from the WHO FCTC (38) indicates that rural districts areas often face weaker infrastructure on account of limited resources for retailer inspections. The 2020 WHO FCTC Needs Assessment highlights the absence of tobacco retail licensing as a critical gap in Lao PDR, noting that licensing is essential for controlling widespread availability of tobacco products. Implementing licensing fees could provide a sustainable local revenue for supporting monitoring and inspection activities (39). Lao PDR currently has adopted the licensing requirement as per WHO FCTC Article 15 (28). Some efforts to further strengthen retail licensing could be made to improve monitoring while also applying penalties for retailers selling non-compliant tobacco products. Future research could focus on conducting longitudinal audits to monitor the enforcement of tobacco control regulations, particularly through targeted interventions in rural areas.

## Data Availability

The original contributions presented in the study are included in the article/Supplementary material, further inquiries can be directed to the corresponding author.
