# Supplementary material for: Tobacco retail characteristics across urban and rural districts in Lao PDR: an observational study, 2024
Source: Front Public Health. 2026 Mar 24;14:1784257. doi: 10.3389/fpubh.2026.1784257 (PMC13055868; doi:10.3389/fpubh.2026.1784257)
Supplement: Supplementary file 1 [file Data_Sheet_1.docx]

**Supplemental Figure 1:** Distribution of tobacco retail outlets within buffers of 250 and 500 m around schools in the urban districts of Chanthabuly and Sissatanak, Lao People’s Democratic Republic, 2024


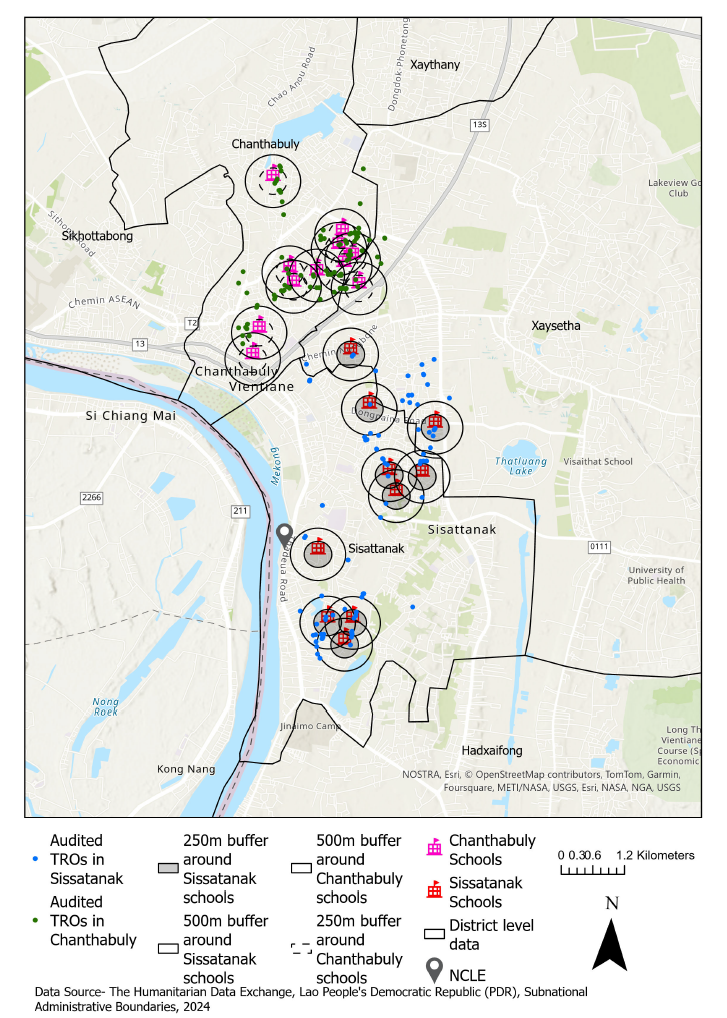


**Supplemental Figure 2**: Distribution of tobacco retail outlets within buffers of 500 and 1000 m around schools in the suburban district of Naxaithong, Lao People’s Democratic Republic, 2024


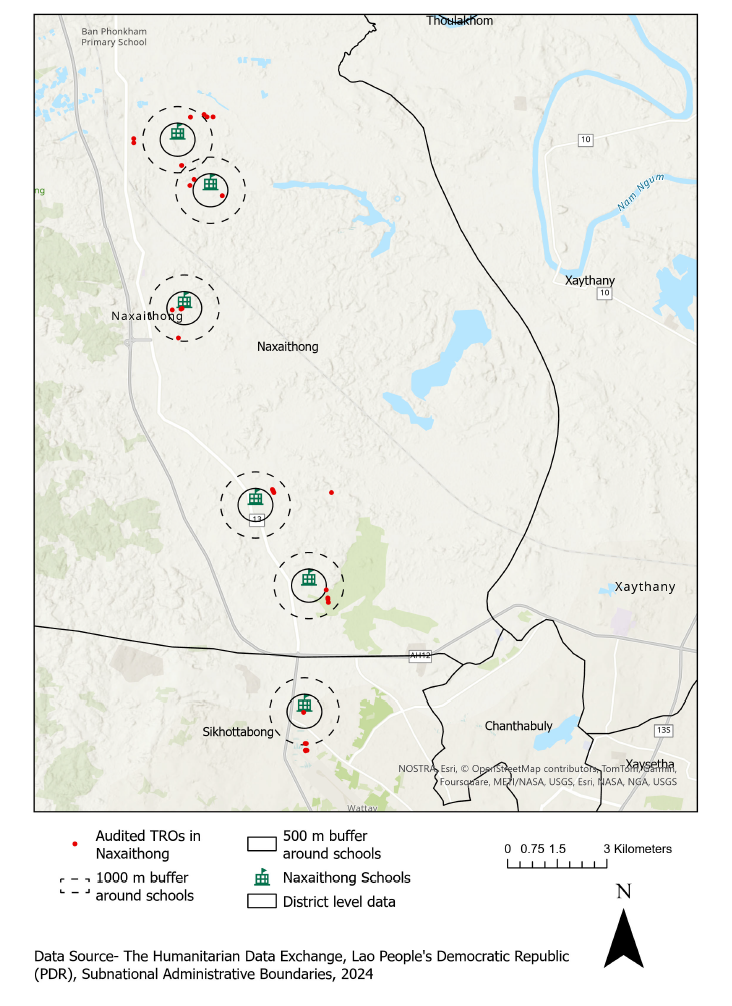


**Supplemental Table 1**: Characteristics of the 27 selected schools (secondary schools and above) in the Chanthabuly, Sissatanak, and Naxaithong districts of Vientiane capital city of Lao PDR, 2024^a^

| Name of the district | Levels of schools | School Name* | Total students | Female students | Male students | Fee structure  (Lao Kip per year) | Annual Fee structure (in USD **) |
| --- | --- | --- | --- | --- | --- | --- | --- |
| Chanthabuly | Secondary school | Sissavath Secondary School | 170 | 90 | 80 | 620,000 | 29.29 |
|  | Secondary school | Phonetong School | 160 | 79 | 81 | 500,000 | 23.60 |
|  | Secondary school | Sinxay Secondary School | 150 | 70 | 80 | 700,000 | 33.05 |
|  | Secondary school | Anou Middle School | 877 | 460 | 417 | 500,000 | 23.60 |
|  | Secondary school | Sengsamai School | 145 | 80 | 65 | 350,000 | 16.52 |
|  | High school | Chanthabuly High School | 256 | 121 | 135 | 800,000 | 37.77 |
|  | High school | Neerada School* | 691 | 357 | 334 | 1,500,000 | 70.86 |
|  | High school | Progress School | 330 | 155 | 175 | 850,000 | 40.13 |
|  | High school | Liewtou Chinese School* | 1182 | 619 | 563 | 1,200,000 | 56.66 |
|  | High school | Sotthachlith School* | 205 | 73 | 132 | 750,000 | 35.43 |
|  | College | Vientiane Professional Development College* | 260 | 50 | 210 | 2,600,000 | 122.84 |
| Sissatanak | Secondary school | Sissatanak Secondary School | 350 | 183 | 167 | 400,000 | 18.88 |
|  | Secondary School | Sharon International School* | 80 | 35 | 45 | 6,258,000 | 295.67 |
|  | Secondary school | Watsop Secondary School | 116 | 47 | 69 | 500,000 | 23.62 |
|  | Secondary school | Phunmun Secondary school | 120 | 54 | 66 | 600,000 | 28.30 |
|  | High school | Saimoungkhoun High School | 167 | 75 | 92 | 450,000 | 21.26 |
|  | High school | Phaiwat School | 801 | 412 | 389 | 350,000 | 16.53 |
|  | High school | Kiettisack International School* | 483 | 226 | 257 | 8,344,000 | 393.63 |
|  | High school | Vientiane International School* | 240 | 97 | 143 | 7,301,000 | 344.43 |
|  | High school | Sengdara International School* | 186 | 62 | 124 | 9,387,000 | 442.84 |
|  | College | Sonephitak Laos College* | 280 | 92 | 188 | 800,000 | 37.77 |
| Naxaithong | Secondary school | Nongniaw School | 236 | 113 | 123 | 350,000 | 16.51 |
|  | Secondary school | Sikeud School | 973 | 506 | 467 | 300,000 | 14.15 |
|  | High school | Naxaithong School | 1468 | 782 | 686 | 250,000 | 11.79 |
|  | High school | Sengsay School | 232 | 106 | 126 | 300,000 | 14.16 |
|  | High school | Ilay School | 1149 | 621 | 528 | 300,000 | 14.16 |
|  | High school | Farseekam School | 412 | 229 | 183 | 250,000 | 11.80 |

^*^ Data source: Lao Ministry of Education (MOE) <http://emisform.lesmis.edu.la:8880/reportview.php?qrid=lse_stu_school&qrtitle> 2024; Lower Secondary school includes students aged 11-15 years (for 4 years); Upper secondary school includes students aged 15-18 years (for 3 years); High school and College includes students aged 18 years and above, ^**^ Lao Kip to USD used the 2024 conversion rate., * suggests schools included as a part of the convenience sample

**Supplemental Table 2:** Classification of TRO type

| **TRO-type** | **Definition** |
| --- | --- |
| Convenience store | Store selling variety of items including snacks, cleaning supplies, medicines, cosmetics, and grocery items along with tobacco products. |
| Supermarket or grocery store | Store selling only grocery items including essential food items along with tobacco products. |
| Roadside kiosk | Any informal roadside shop selling predominantly tobacco products. |
| Restaurants having informal outlets | Informal outlets selling few tobacco products besides serving food as primary business. |
| Others | Other businesses such as electronic stores, or stores offering mobile recharge services, selling fruit juices, selling footwear, stores as a part of market selling vegetables, or stores with car/bike rental services also selling tobacco products. |

**Supplemental Figure 2A**: Tobacco retail outlets across the urban district of Chanthabuly, Lao PDR, 2024

The **top left** photo shows a ‘We ID’ sign on the cabinet showcasing cigarette packs organized neatly on the cabinet near the store entrance (marked by green arrow) at a convenience store; **the top right** photo also shows a ‘We ID’ sign (marked by green arrow) on the cabinet selling cigarette packs; the **bottom photo** shows a roadside kiosk selling cigarette packs next to eggs.


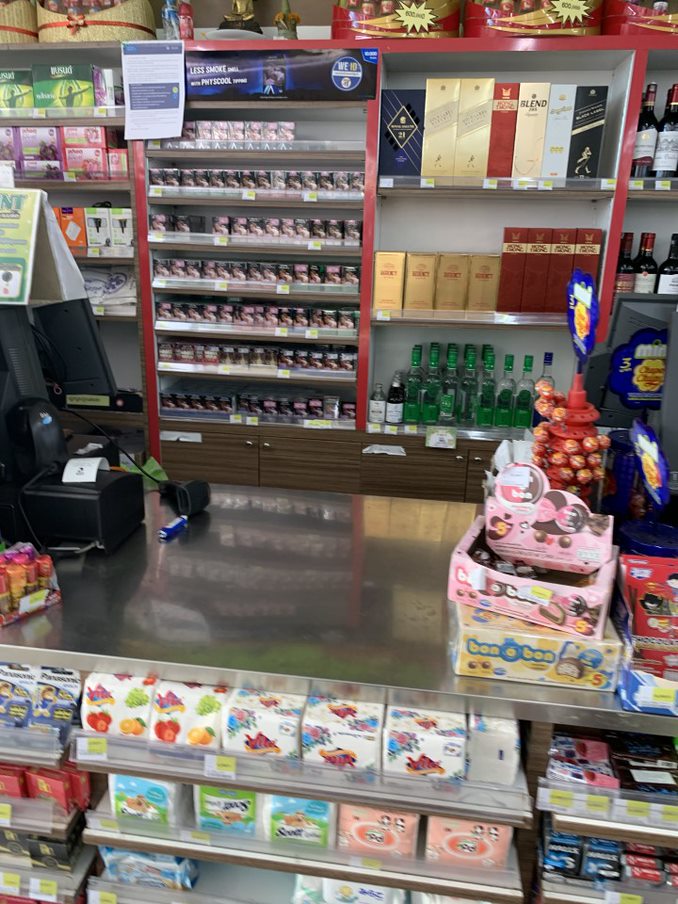

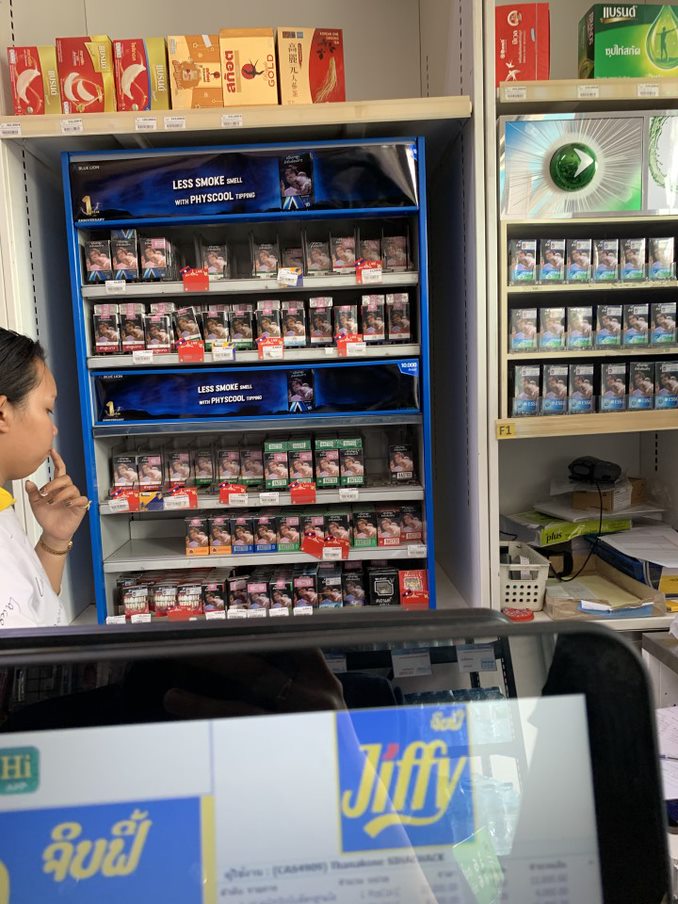

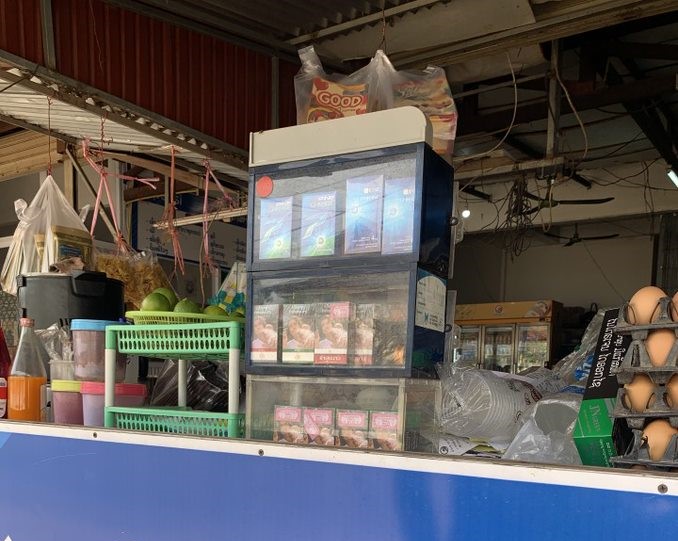


**Supplemental Figure 2B:** Tobacco retail outlets across the urban district of Sissatanak, Lao PDR, 2024


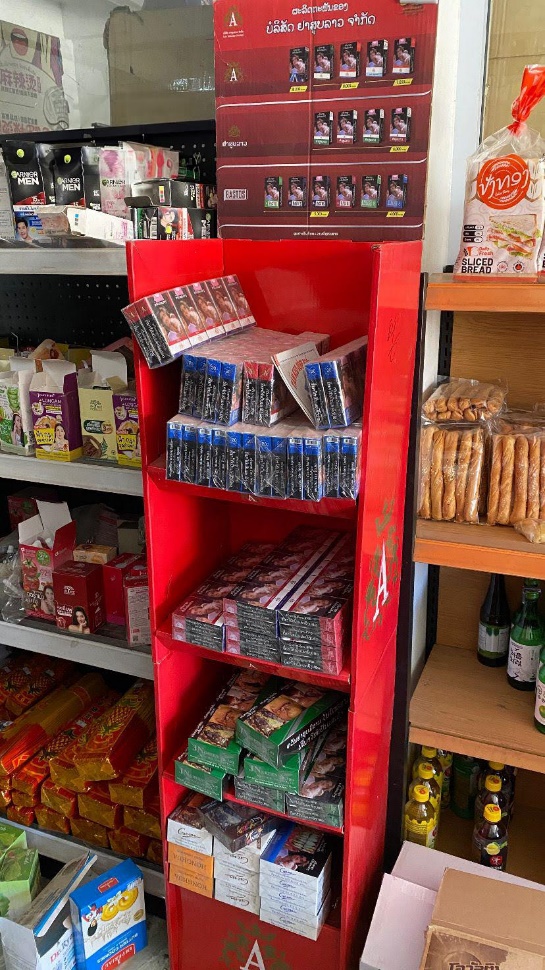

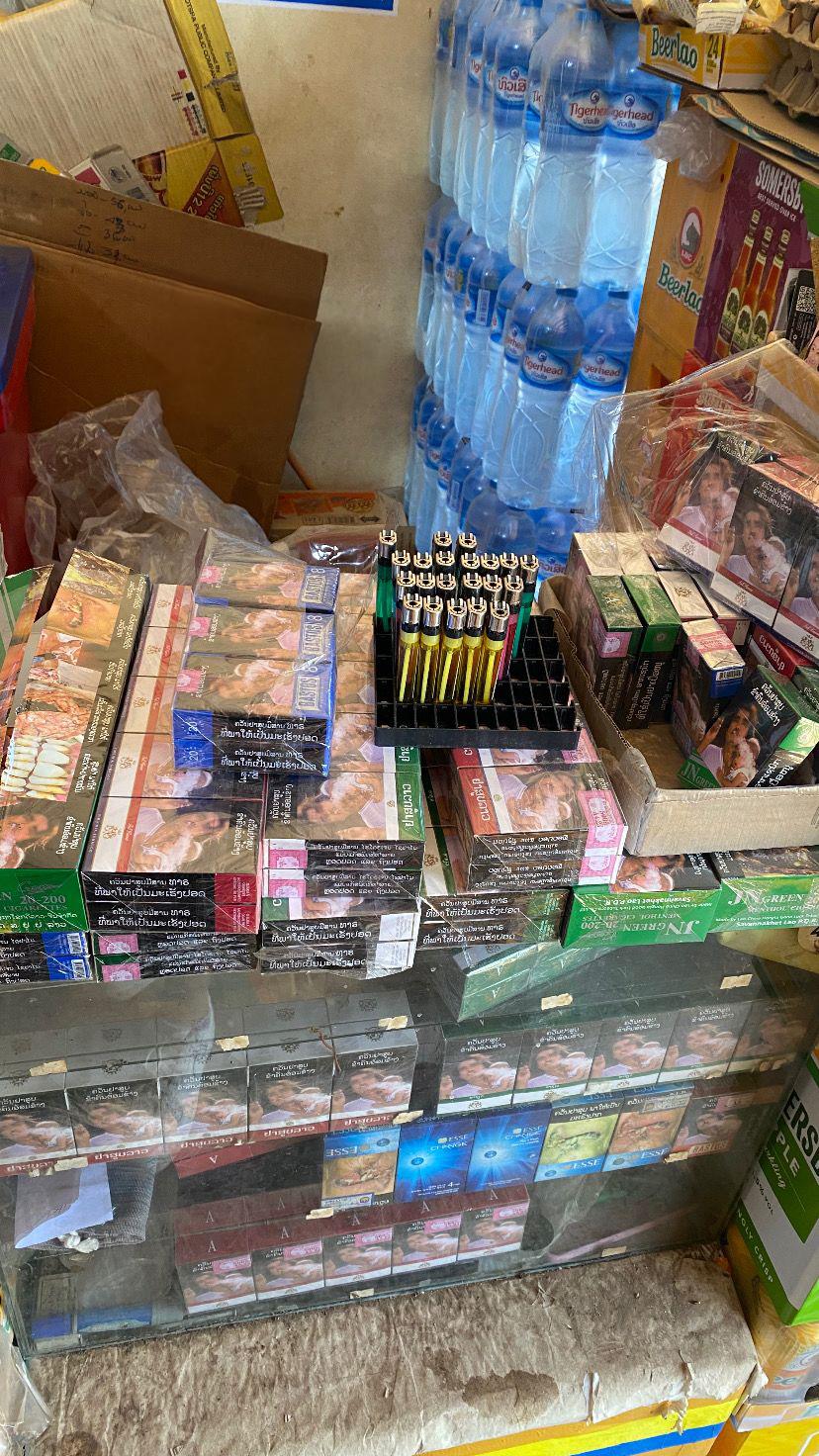


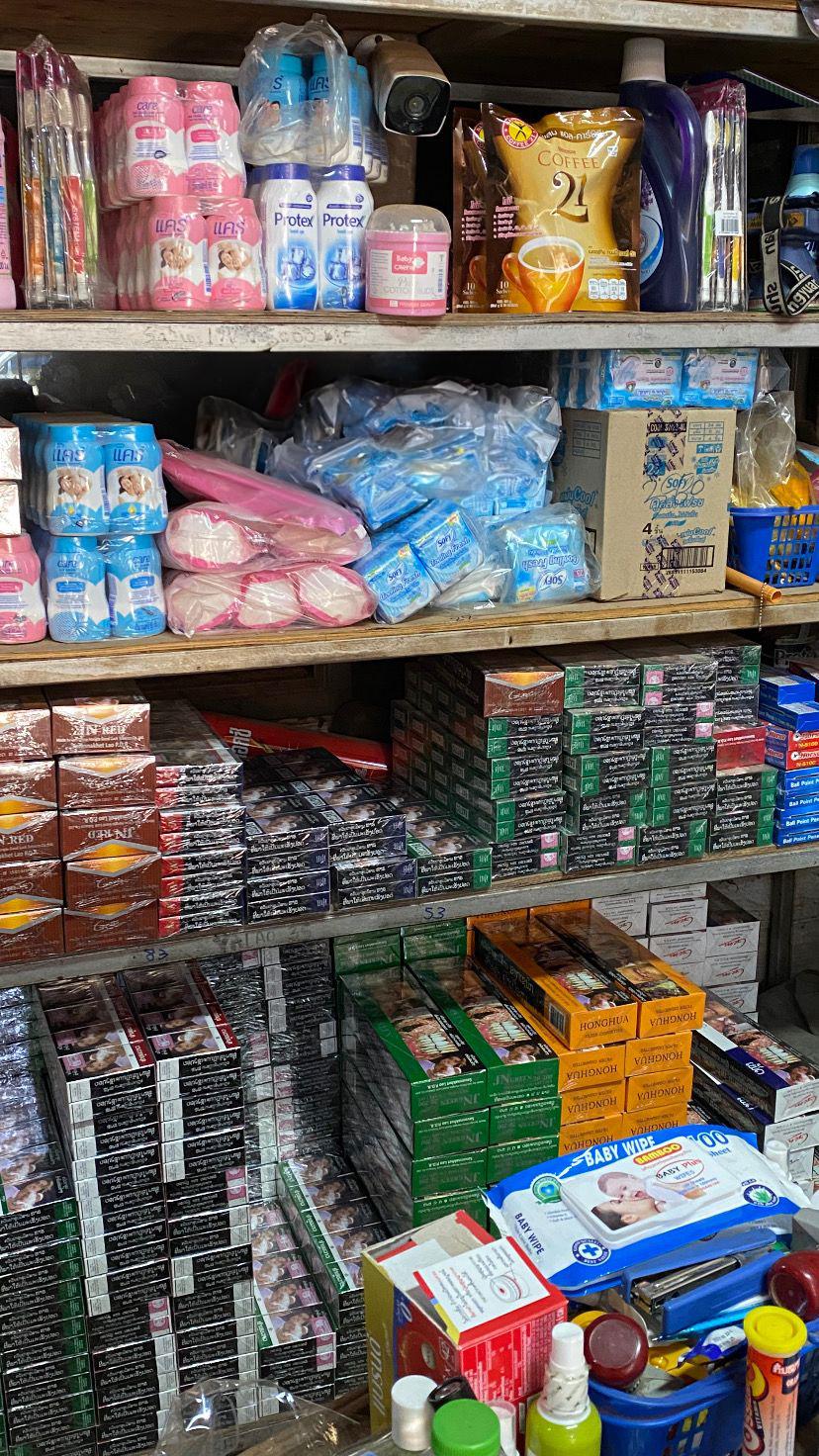


The **top left** photo shows “Lao Tobacco Limited’ printed in red color on the cabinet (marked by a yellow arrow) showcasing the cigarette packs at a supermarket organized neatly on the cabinet; the **top right** photo shows a variety of cigarette packs being displayed at a supermarket entrance along with the sale of lighters; **bottom photo** shows grocery items such as coffee, tissues, milk powder being sold next to cigarette packs in a supermarket.


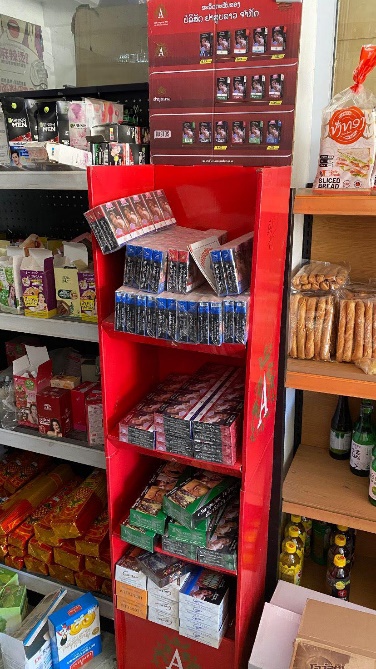


Top left picture shows “Lao Tobacco Limited’ printed in red color on the cabinet (marked by yellow arrow) showcasing the cigarette packs at a supermarket organized neatly on the cabinet; top right picture shows a variety of cigarette packs being displayed at a supermarket entrance along with sale of lighters; bottom picture shows grocery items such as coffee, tissues, milk powder being sold next to cigarette packs in a supermarket.

**Supplemental Figure 2C:** Tobacco retail outlets across the suburban district of Naxaithong, Lao PDR, 2024

Top left photo shows roadside kiosk selling cigarette packs next to beer cans, and lighters; top right photo shows a convenience store selling cigarette packs inside red cabinet marked ‘Lao Tobacco limited’ with lighters sold next to them (marked by green arrows); bottom photo shows a roadside kiosk selling fruits, fruit juices, and lighters next to the cabinet showcasing cigarette packs.


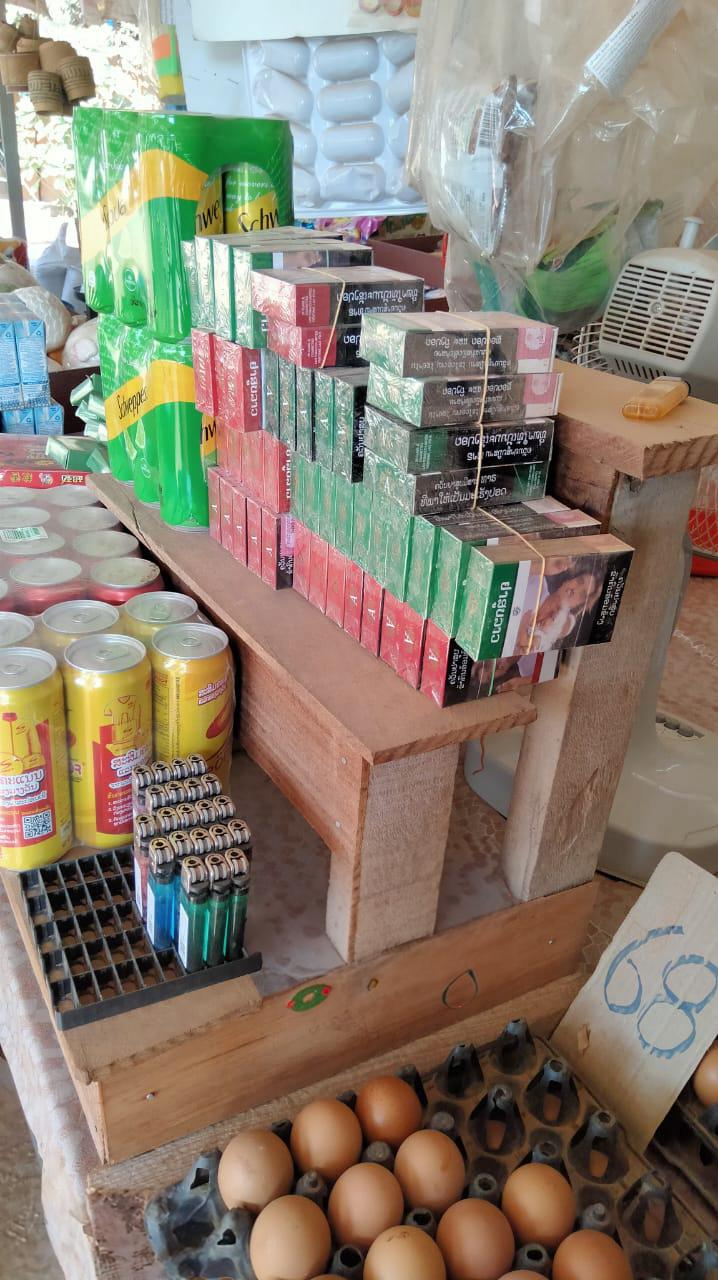

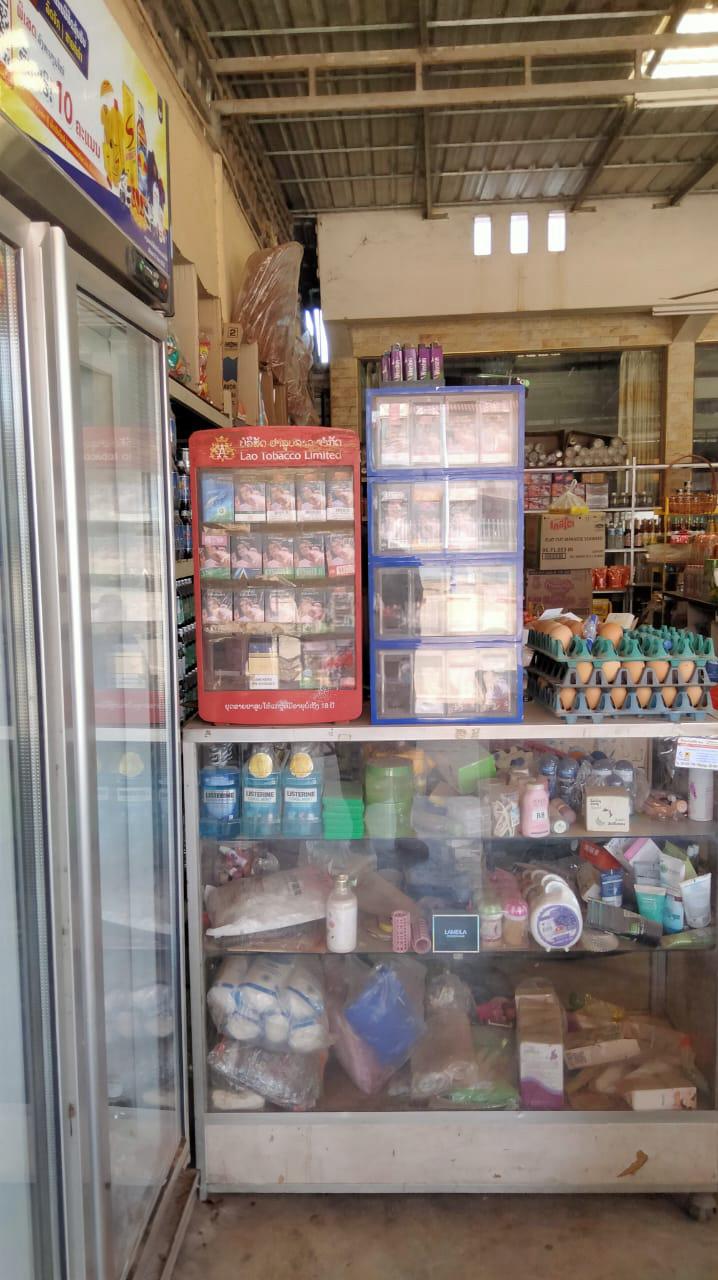


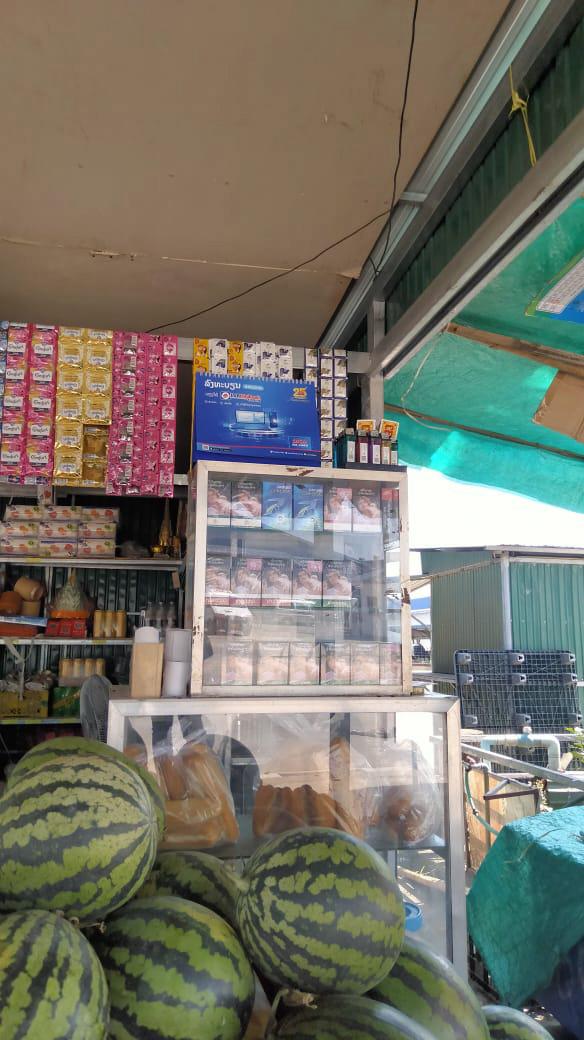


The **top left** photo shows a roadside kiosk selling cigarette packs next to beer cans, and lighters; the **top right photo** shows a convenience store selling cigarette packs inside a red cabinet marked ‘Lao Tobacco limited’ with lighters sold next to them (marked by green arrows**); bottom photo** shows a roadside kiosk selling fruits, fruit juices, and lighters next to the cabinet showcasing cigarette packs.
